# Development of a Supercritical Fluid Chromatography-Tandem Mass Spectrometry Method for the Determination of Azacitidine in Rat Plasma and Its Application to a Bioavailability Study

**DOI:** 10.3390/molecules19010342

**Published:** 2013-12-27

**Authors:** Dongpo Li, Tianhong Zhang, Longfa Kou, Youxi Zhang, Jin Sun, Zhonggui He

**Affiliations:** 1 Department of Pharmaceutics, Shenyang Pharmaceutical University, 103 Wenhua Road, Shenyang 110016, China; E-Mails: ldcover@163.com (D.L.); klf.666666@163.com (L.K.); zyx5131410@163.com (Y.Z.); 2 Department of Pharmaceutical Analysis, Shenyang Pharmaceutical University, 103 Wenhua Road, Shenyang 110016, China; E-Mail: pharmazhang@163.com

**Keywords:** azacitidine, SFC-MS/MS, rat plasma, intravenous

## Abstract

Azacitidine is widely used for the treatment of myelodysplastic syndromes (MDS) and acute myelogenous leukaemia (AML). The analysis of azacitidine in biological samples is subject to interference by endogenous compounds. Previously reported high-performance liquid chromatography/tandem mass spectrometric (HPLC-MS/MS) bioanalytical assays for azacitidine suffer from expensive sample preparation procedures or from long separation times to achieve the required selectivity. Herein, supercritical fluid chromatography with tandem mass spectrometry (SFC-MS/MS) was explored as a more promising technique for the selective analysis of structure-like or chiral drugs in biological matrices. In this study, a simple, rapid and specific SFC/MS/MS analytical method was developed for the determination of azacitidine levels in rat plasma. Azacitidine was completely separated from the endogenous compounds on an ACQUITY UPLC™ BEH C_18_ column (100 mm × 3.0 mm, 1.7 μm; Waters Corp., Milford, MA, USA) using isocratic elution with CO_2_/methanol as the mobile phase. The single-run analysis time was as short as 3.5 min. The sample preparation for protein removal was accomplished using a simple methanol precipitation method. The lower limit of quantification (LLOQ) of azacitidine was 20 ng/mL. The intra-day and inter-day precisions were less than 15%, and the relative error (RE) was within ±15% for the medium- and high-concentration quality control (QC) samples and within ±20% for the low-concentration QC samples. Finally, the developed method was successfully applied to a pharmacokinetic study in rats following the intravenous administration of azacitidine.

## 1. Introduction

**Figure 1:**
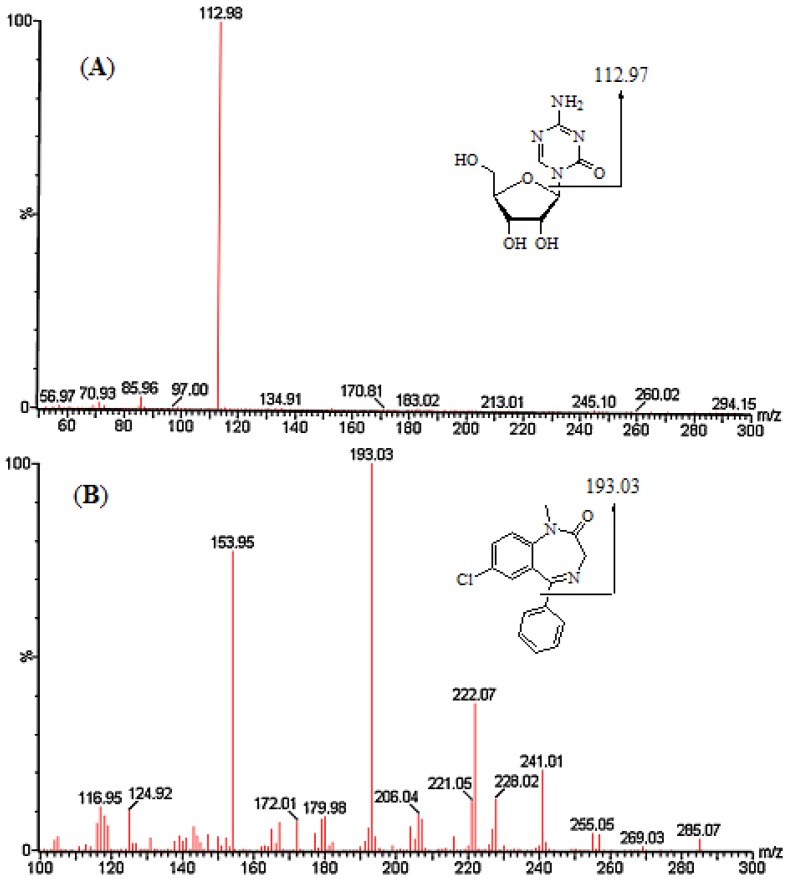
Product ion mass spectra of [M+H]^+^ ions of azacitidine (**A**) and diazepam (**B**).

Myelodysplastic syndromes (MDS) represent a diverse group of haematopoietic disorders derived from the inefficient production of blood cells, each with varying risks of transformation to acute myelogenous leukaemia (AML) [1,2]. Azacitidine (AC, Figure 1A) was developed as a novel DNA methyltransferase inhibitor, and it has been approved by the FDA for the treatment of MDS [3,4]. Additionally, azacitidine has been found to exhibit significant clinical benefits in AML [5,6,7].

Azacitidine is unstable and is rapidly hydrolysed to several degradation products, including 5-azacytosine and 5-azauracil [8]. Azacitidine has a narrow therapeutical window, and an accurate quantitative method that would permit the monitoring of its plasma levels to ensure that these levels are maximally therapeutic but minimally toxic is urgently needed. The bioanalysis of azacitidine is subject to interference from endogenous compounds. Originally, microbiological and radioactive carbon-labelling assays were used to determine azacitidine levels in biological samples, but the assays were not specific or accurate [9,10]. Early high-performance liquid chromatography (HPLC) assays were developed to quantitate azacitidine in aqueous media but not in plasma, and the data only confirmed previous assumptions concerning the hydrolytic kinetics [8,11,12]. HPLC assays have subsequently been established for the determination of azacitidine levels in biological fluids, but these assays suffer from low sensitivity, with a lower limit of quantification (LLOQ) of 250 ng/mL [13]. Recently, an HPLC-tandem mass spectrometry (HPLC-MS/MS) method was developed to completely separate azacitidine from the endogenous compounds in plasma samples, but the technique was time consuming (30 min) and expensive (solid-phase extraction) [14]. The popularity of supercritical fluid chromatography (SFC)-MS/MS methods for enantiomeric separations has increased over the past few years [15]. SFC-MS/MS methods have been found to provide good retention, symmetrical peak shapes and sufficient separation from matrix constituents. This study intended to develop an SFC-MS/MS method for the separation of azacitidine and the endogenous substances in rat plasma samples. Compared with the previously reported methods, this validated method resulted in a shorter analytical time (3.5 min per sample), a smaller blood volume (50 μL) and a convenient extraction method to completely dissociate azacitidine from blood samples with an LLOQ of 20 ng/mL. To our knowledge, this paper is the first report that establishes an efficient and inexpensive method for the complete separation of endogenous substances in combination with an SFC-MS/MS method for the determination of azacitidine levels in blood samples. Finally, the validated method was applied to a preliminary pharmacokinetic study in rats following the intravenous administration of azacitidine.

## 2. Results and Discussion

### 2.1. Method Development

During the optimisation of the mass spectrometric parameters, the chemical structures (Figure 1) of azacitidine and diazepam (internal standard, IS) indicated that they captured a proton and produced a strong mass response in positive ionisation mode. The identification of the precursor ions and product ions for azacitidine and diazepam is presented in Figure 1. The multiple-reaction monitoring (MRM) transitions of azacitidine and diazepam were *m/z* 244.92→112.97 and 285.07→193.03, respectively.

The chemical structure of azacitidine is similar to that of other high-concentration endogenous compounds in rat plasma. Therefore, sufficient chromatographic separation of azacitidine from the endogenous compounds is difficult to accomplish in biological samples using ultra-performance liquid chromatography (UPLC)-MS/MS assays. Based on previously published HPLC-MS/MS methods, HPLC columns have been utilised to retain the analytes under a complex gradient elution conducted for chromatographic separation [14]. Though the HPLC-MS/MS methodology was reliable, accurate and precise, it required a long 30 min separation to yield adequate resolution between azacitidine and the endogenous compounds.

In this work, we investigated the potential of an SFC-MS/MS method as a complimentary approach to the HPLC-MS/MS method for the determination of azacitidine levels in rat plasma samples. The low-viscosity and highly diffuse SFC mobile phase has the potential for fast separations at higher flow rates. The SFC separation of azacitidine from the endogenous compounds was governed by an ethylene bridged hybrid (BEH) C_18_ column using CO_2_/methanol as the mobile phase. An isocratic chromatographic elution was used with an optimised composition of the supercritical CO_2_ fluid and the modifier, methanol, which provided selective separations within a shorter run time of 3.5 min.

A number of methods were assessed to efficiently extract the drugs from the plasma, including simple protein precipitation and solid-phase extraction with a variety of diluents. As mentioned above, solid-phase extraction procedures have been described as sample pre-treatments [14]. However, this procedure is time consuming and expensive. Simple protein precipitation using methanol or acetonitrile has been commonly employed to extract similarly structured drugs from biological samples [16]. Therefore, a protein precipitation sample preparation technique using methanol was optimised, and the recovery was high and reproducible.

### 2.2. Method Validation

**Figure 2:**
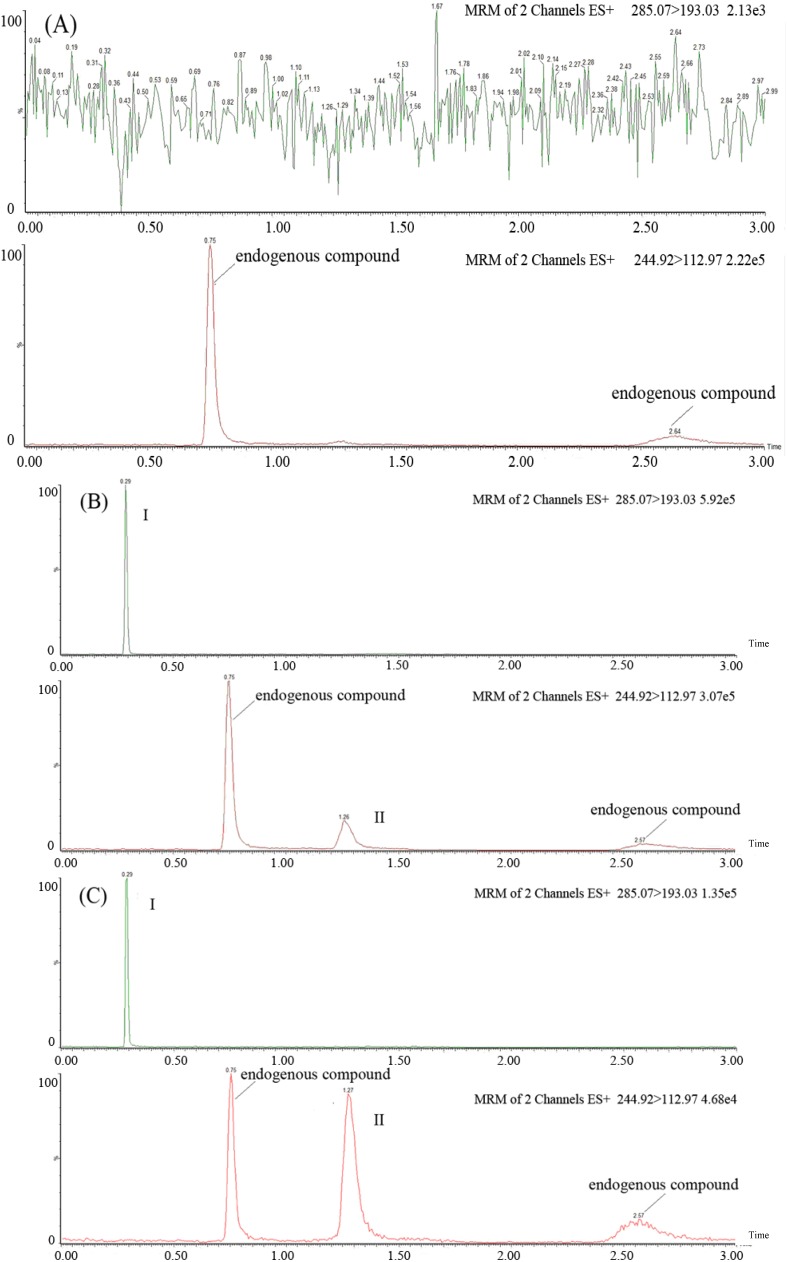
Representative MRM chromatograms of diazepam (IS, I) and azacitidine (II) in rat plasma: (**A**) a blank rat plasma sample; (**B**) a blank rat plasma sample spiked with azacitidine (20 ng/mL) and diazepam (2,000 ng/mL); (**C**) a rat plasma sample following intravenous administration of azacitidine at 2.5 mg/kg to Sprague-Dawley rats.

Six control samples prepared from different rat plasma samples were analysed to evaluate the presence of interference using the described SFC-MS/MS assay. Representative chromatograms of blank plasma, blank plasma spiked with azacitidine (20 ng/mL) and diazepam (2,000 ng/mL), and plasma samples following intravenous administration are shown in Figure 2. It was evident that there was no interference from endogenous substances in the plasma at the retention times of the analytes and IS.

**Table 1:** Matrix effects and recoveries of azacitidine and diazepam (IS) in rat plasma after samplepreparation. The data are presented as means ± S.D. (n = 6). Matrix effect (%) = (SET 2/SET 1) × 100; Recovery (%) = (SET 3/SET 2) × 100; SET 1: the peak areas obtained from neat solution standards; SET 2: thepeak areas from standards spiked after extraction; SET 3: the peak areas from standards spiked prior to extraction.

Nominal Concentration (ng/mL)		Peak area			Matrix effect (%)	Recovery (%)
		SET 1	SET 2	SET 3		
Plasma azacitidine	20	337 ± 21	270 ± 22	214 ± 9	80.3	79.2
	2,000	53,115 ± 1,275	43,340 ± 2,915	36,507 ± 1,394	81.6	84.2
	10,000	256,686 ± 2,592	235,618 ± 13,942	202,066 ± 10,854	91.8	85.8
Diazepam (IS)	2000	62,114 ± 1,497	55,308 ± 3,698	47,741 ± 2,797	89.0	86.3

The extraction recoveries in the rat plasma for azacitidine were determined at three levels of quality control (QC) samples: 20, 2,000 and 10,000 ng/mL. The extraction recovery of diazepam was also evaluated. The mean extraction recoveries were 79.2%, 84.2% and 85.8% at concentrations of 20, 2,000 and 10,000 ng/mL, respectively, for azacitidine. The extraction recovery of IS was 86.3%. The ratios of the matrix effects for azacitidine and IS ranged from 85% to 115%, suggesting that there were no significant matrix effects using this method. All the data are shown in Table 1.

Calibration curves were plotted between the peak area ratio of the analyte and internal standard and the nominal concentration of the analyte. The 1/x^2^ weighted least-squares linear regression was fitted over the 20–20,000 ng/mL range for azacitidine. The typical equation for the calibration curve was as follows: azacitidine, y = 0.199x − 4.41. The correlation coefficients (r) exceeded 0.99, showing good linearity over the concentration range. The LLOQ was 20 ng/mL for azacitidine in rat plasma. 

Table 2 summarises the intra-day and inter-day precision and accuracy for azacitidine from the QC samples. The results indicated that all the values were within the acceptable range of ±15% for the medium- and high-concentration QC samples and ±20% for the low-concentration QC samples, and the method exhibited good precision and accuracy.

**Table 2:** Accuracy and precision forthe analysis of azacitidine in rat plasma (three validation days, six replicates at each concentration level per day).

Concentration (ng/mL)		RSD (%)		RE (%)
Added	Found	Intra-day	Inter-day	
20	18.6	5.9	18.3	−7.0
2,000	2,074.5	6.8	10.6	3.7
10,000	10,014.8	8.8	9.0	0.2

A number of stability experiments were performed, and the results are shown in Table 3. No significant changes in the azacitidine concentrations were observed under the indicated conditions.

**Table 3:** Stability of azacitidine in rat plasma exposed to various storage conditions (n = 3).

Storage conditions	Concentration (ng/mL)		RSD (%)	RE (%)
	Added	Found		
Ambient, 2 h	20	24.0	3.4	20.0
	10,000	10,388.5	6.0	3.9
−80 °C, 30 days	20	24.0	1.8	20.0
	10,000	9,519.1	6.7	−4.8
Three freeze–thaw cycles	20	24.0	2.5	20.0
	10,000	10,073.4	10.6	0.7
Sample rack for 24 h at 4 °C	20	23.8	1.8	19.0
	10,000	9,966.3	8.7	−0.3

### 2.3. Pharmacokinetic Application of the Method

**Figure 3:**
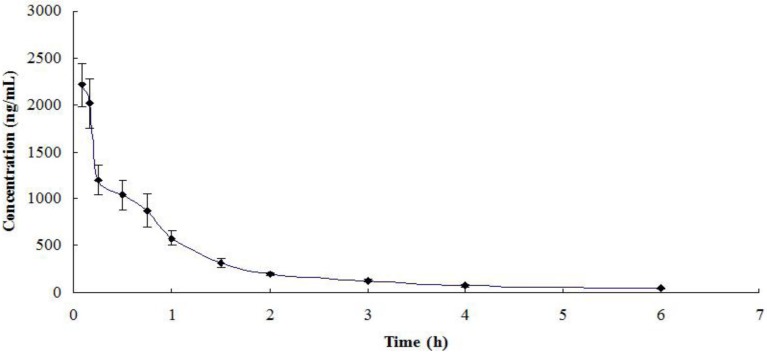
Mean plasma concentration-time profile of azacitidine following intravenous administration at 2.5 mg/kg to Sprague-Dawley rats (n = 3).

The presented method was applied to quantify azacitidine following intravenous administration at a single dose of 2.5 mg/kg to Sprague-Dawley (SD) rats. The mean plasma concentration-time profile is shown in Figure 3. The pharmacokinetic parameters are listed in Table 4. The AUC_0-∞_ of azacitidine in the plasma was 1996.0 ng·h/mL, and the elimination half-life (*t*_1/2_) was 0.506 h.

**Table 4:** Main pharmacokinetic parameters of azacitidine in rats following intravenous administration at a dose of 2.5 mg/kg (mean ± SD, n = 3).

Parameters	Azacitidine
t_1/2β_ (h)	0.506 ± 0.198
AUC _0-∞_ (μg·h/mL)	1996.0 ± 198.7
V_d_ (L/kg)	2.07 ± 0.17
CL (L/h/kg)	1.253 ± 0.085

## 3. Experimental

### 3.1. Chemicals and Reagents

Azacitidine and diazepam (internal standard, IS, 99.9% purity) were purchased from the National Institute for Control of Pharmaceutical and Biological Products (Beijing, China). Tetrahydrouridine (THU), a deaminase inhibitor, was purchased from Calbiochem (La Jolla, CA, USA). HPLC-grade methanol was purchased from Fisher Scientific (Pittsburgh, PA, USA). Triple deionised water was obtained using an Easypure^®^ II RF/UV ultra-pure water system (Barnstead International Corp., Dubuque, IA, USA).

### 3.2. Instrumentation

A Waters ACQUITY Tandem Quadrupole Detector (TQD) system was employed for the determination of the analytes of interest, which consisted of an ACQUITY UPC system with a cooling autosampler and column oven and an ACQUITY triple quadrupole tandem mass spectrometer with an electrospray ionisation (ESI) interface (Waters Corp., Milford, MA, USA). A BEH C_18_ column (100 mm × 3.0 mm, 1.7 μm, Waters Corp., Wexford, Ireland) was used to separate the analytes. All data were acquired and processed using MassLynx 4.1 software with the QuanLynx program (Waters Corp).

### 3.3. SFC/MS/MS Condition

The separation of azacitidine from the endogenous compounds in rat plasma by supercritical fluid chromatography was conducted using an isocratic mobile phase composed of CO_2_/methanol. All samples were run in an 80% CO_2_ mobile phase at 2.5 mL/min. The analytical column was maintained at 45 °C, and the back pressure was controlled at 1,500 psi. The autosampler was conditioned at 4 °C, and the sample volume injected was 2 µL.

A Waters TQD with an ESI source operating in the positive ionisation mode was used to detect azacitidine and diazepam. The MS/MS conditions were as follows: capillary voltage 3.0 kV, source temperature 120 °C and desolvation temperature 550 °C. The optimised collision energy was 14 and 33 eV for azacitidine and diazepam, respectively, and the cone voltage was 16 and 47 eV, respectively. Under these SFC-MS/MS conditions, the precursor/product ion transitions were *m/z* 244.92/112.97 for azacitidine and *m/z* 285.07/193.03 for diazepam. The scan time was set at 0.02 s per transition.

### 3.4. Preparation of Standard and QC Samples

Stock solutions of azacitidine and diazepam (IS) were prepared individually at concentrations of 1 mg/mL and 2,000 ng/mL in methanol, respectively. The standard solutions of azacitidine were prepared by serially diluting the stock solution of azacitidine with methanol. The calibration standards were prepared at concentrations of 20, 50, 100, 200, 500, 1,000, 2,000, 5,000, 7,500, 10,000 and 20,000 ng/mL. All the solutions were stored at −20 °C and brought to room temperature before use.

The analyte standard solutions were spiked in blank rat plasma containing THU (0.1 mM), followed by the addition of IS solution to obtain calibration standards in the concentration range of 20 ng/mL to 20 µg/mL for azacitidine. The QC samples were prepared to contain 20, 2,000 or 10,000 ng/mL azacitidine and 2000 ng/mL diazepam in the same manner as the calibration samples.

### 3.5. Plasma Sample Preparation

After thawing at room temperature, 50 μL of the IS solution (2,000 ng/mL) and 100 μL methanol were added to a 50 μL aliquot of plasma sample. The protein in the plasma samples was precipitated by methanol. The samples were then vortexed for 3 min and centrifuged at approximately 13,000 rpm for 10 min. The upper organic layer was removed and injected by the autosampler into all hyphenated MS systems for quantitative analysis.

### 3.6. Method Validation

The selectivity was assessed by comparing the chromatograms of six different batches of blank rat plasma with the corresponding spiked rat plasma. The extraction recoveries were calculated as the ratio of the analyte peak areas from the extracted QC samples to the mean peak areas of the extraction samples post-spiked with azacitidine and diazepam. The matrix effects for the azacitidine and diazepam were evaluated by comparing the peak areas of the analytes in the post-extraction spiked blank plasma at QC concentrations with the peak areas for the corresponding standard solutions. The linearity was assessed by weighted (1/x^2^) least-squares analysis of six different calibration curves. The LLOQ of the assay was the lowest concentration of the analyte with the recommended precision and accuracy less than 20%. The intra- and inter-day precision (the relative standard deviation, RSD) and accuracy (the relative error, RE) were determined by the analysis of low, medium, and high QC samples (n = 6) on 3 different days. The stability of the QC samples (n = 3) in three complete freeze/thaw cycles (−80 to 20 °C), long-term sample storage (−80 °C for 30 days) and on the bench top (20 °C for 2 h) was assessed. The ready-for-injection stability of the extracted samples in the autosampler rack at 4 °C for 24 h was also evaluated. The analytes were considered stable when the accuracy bias was within ± 20% of the nominal concentration.

### 3.7. Pharmacokinetic (PK) Study in Rats

Male SD rats weighing from 210 to 240 g were used for the PK study. All animal experiments were performed in accordance with institutional guidelines and were approved by the University Committee on Use and Care of Animals, Shenyang Pharmaceutical University. An aqueous solution of azacitidine was administered to the rats by tail vein injection at a dose of 2.5 mg/kg. Serial blood samples (0.2 mL) were obtained at 0.08, 0.17, 0.25, 0.5, 0.75, 1, 1.5, 2, 3, 4, 6, 8, 10 and 12 h after intravenous administration. All samples were placed into heparinised tubes. The rat plasma samples were centrifuged at 13,000 rpm for 10 min, and the plasma supernatant was collected. THU was added to the plasma supernatant at a final concentration of 100 µM. The plasma was frozen at −80 °C until analysis.

## 4. Conclusions

To date, a number of publications have reported the determination of azacitidine concentrations, but no comprehensive study of the determination of azacitidine levels with a complete separation of endogenous substances in biological samples has yet been performed. To achieve the complete separation of endogenous substances and the accurate determination of azacitidine levels in blood samples, a single-step protein precipitation method in combination with an SFC-MS/MS method was established and optimised for studying the pharmacokinetic profiles of azacitidine. The SFC-MS/MS method was developed and validated for the determination of azacitidine levels in rat plasma from 20 to 20,000 ng/mL. The CO_2_/methanol normal-phase SFC was able to provide good separation efficiency between azacitidine and endogenous substances. The method had the advantage of a total analysis time of 1/10 that of the previously reported HPLC-MS/MS methodology, with a single analysis time of 3.5 min/per sample. In addition, this method showed easy sample preparation and the complete separation of the endogenous substances, and it could be easily extended to other biological matrices. The applicability of the method was demonstrated in a pharmacokinetic study of azacitidine in rats following intravenous administration.
